# Crystal structure of ethyl 2-phenyl-4-(prop-2-yn-1-yl­oxy)-5,6,7,8-tetra­hydro­pyrido[4′,3′:4,5]thieno[2,3-*d*]pyrimidine-7-carboxyl­ate

**DOI:** 10.1107/S2056989015018447

**Published:** 2015-10-10

**Authors:** Mehmet Akkurt, Victoria A. Smolenski, Shaaban K. Mohamed, Jerry P. Jasinski, Essam K Ahmed, Mustafa R. Albayati

**Affiliations:** a Department of Physics, Faculty of Sciences, Erciyes University, 38039 Kayseri, Turkey; b Department of Chemistry, Keene State College, 229 Main Street, Keene, NH 03435-2001, USA; c Chemistry and Environmental Division, Manchester Metropolitan University, Manchester M1 5GD, England; d Chemistry Department, Faculty of Science, Minia University, 61519 El-Minia, Egypt; e Kirkuk University, College of Science, Department of Chemistry, Kirkuk, Iraq

**Keywords:** crystal structure, thieno-pyrimidine, tetra­hydro­pyridine ring, hydrogen bonding, C—H⋯π inter­actions

## Abstract

In the title compound, C_21_H_19_N_3_O_3_S, the 5,6,7,8-tetra­hydro­pyridine ring adopts a half-chair conformation. The fused-thieno[2,3-*d*]pyrimidine ring system is essentially planar (r.m.s. deviation = 0.001 Å) and forms a dihedral angle of 2.66 (6)° with the attached phenyl ring. The three-dimensional crystal packing is stabilized by C—H⋯O and C—H⋯N hydrogen bonds and C—H⋯π inter­actions.

## Related literature   

For general chemistry background to heterocyclic thieno[2,3-*d*]pyrimidines, see: Litvinov (2004 ▸). For the diversity of biological activities of thieno-pyrimidine derivatives, see: Nasr & Gineinah (2002 ▸); Bhuiyan *et al.* (2005 ▸); Chambhare *et al.* (2003 ▸); Alagarsamy *et al.* (2006 ▸). Kapustina *et al.* (1992 ▸). For related structures, see: Liu *et al.* (2005 ▸); Ren *et al.* (2006 ▸).
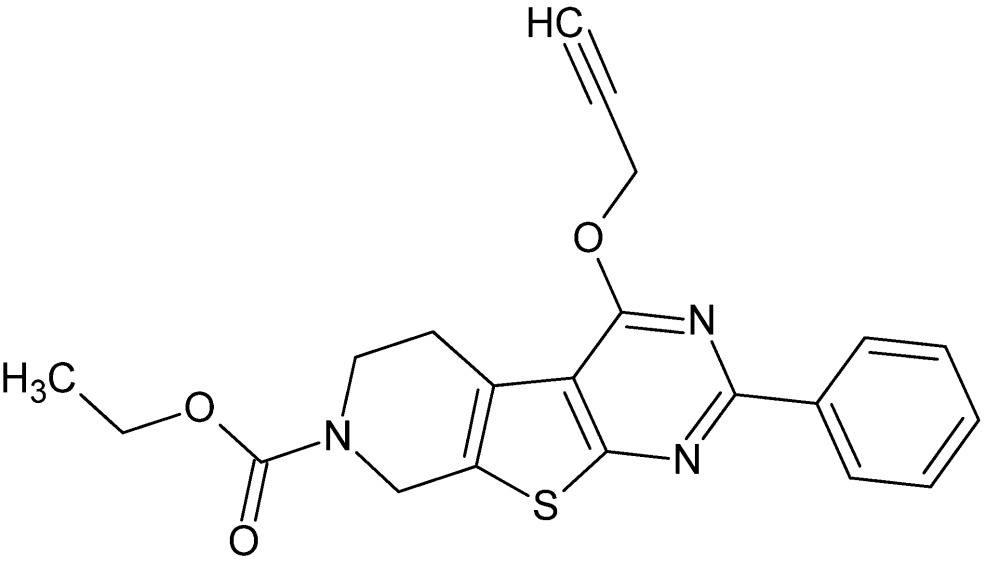


## Experimental   

### Crystal data  

C_21_H_19_N_3_O_3_S
*M* *_r_* = 393.45
Monoclinic, 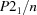

*a* = 13.143 (2) Å
*b* = 8.013 (2) Å
*c* = 17.880 (2) Å
β = 96.129 (14)°
*V* = 1872.3 (6) Å^3^
*Z* = 4
Mo *K*α radiation
μ = 0.20 mm^−1^
*T* = 296 K
0.28 × 0.14 × 0.08 mm

### Data collection  

Agilent Xcalibur, Eos, Gemini diffractometer
Absorption correction: multi-scan (*CrysAlis PRO*; Agilent, 2014 ▸) *T*_min_ = 0.834, *T*_max_ = 1.000
14345 measured reflections
6319 independent reflections
4721 reflections with *I* > 2σ(*I*)
*R*_int_ = 0.032

### Refinement  

*R*[*F* ^2^ > 2σ(*F* ^2^)] = 0.044
*wR*(*F* ^2^) = 0.117
*S* = 1.04
6319 reflections
254 parameters
H-atom parameters constrained
Δρ_max_ = 0.35 e Å^−3^
Δρ_min_ = −0.31 e Å^−3^

### 

Data collection: *CrysAlis PRO* (Agilent, 2014 ▸); cell refinement: *CrysAlis PRO*; data reduction: *CrysAlis PRO*; program(s) used to solve structure: *SHELXS2014* (Sheldrick, 2008 ▸); program(s) used to refine structure: *SHELXL2014* (Sheldrick, 2015 ▸); molecular graphics: *ORTEP-3 for Windows* (Farrugia, 2012 ▸); software used to prepare material for publication: *PLATON* (Spek, 2009 ▸).

## Supplementary Material

Crystal structure: contains datablock(s) global, I. DOI: 10.1107/S2056989015018447/tk5390sup1.cif

Structure factors: contains datablock(s) I. DOI: 10.1107/S2056989015018447/tk5390Isup2.hkl

Supporting information file. DOI: 10.1107/S2056989015018447/tk5390Isup3.cml

**Fig. 1.**
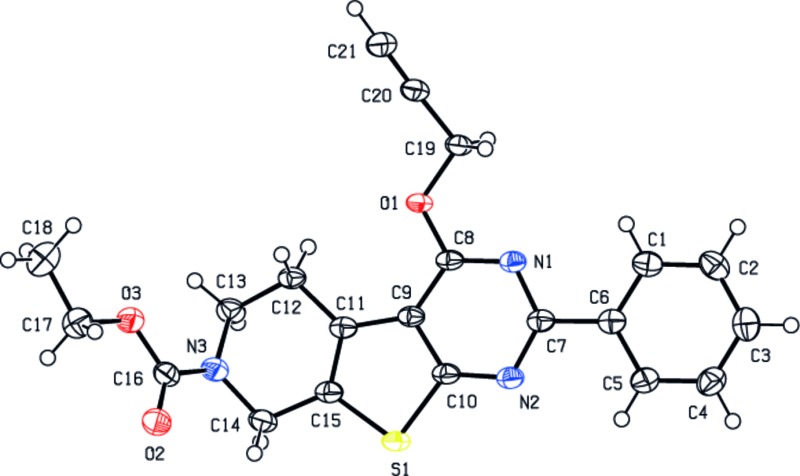
View of the title compound with the atom numbering scheme. Displacement ellipsoids for non-H atoms are drawn at the 50% probability level.

**Fig. 2.**
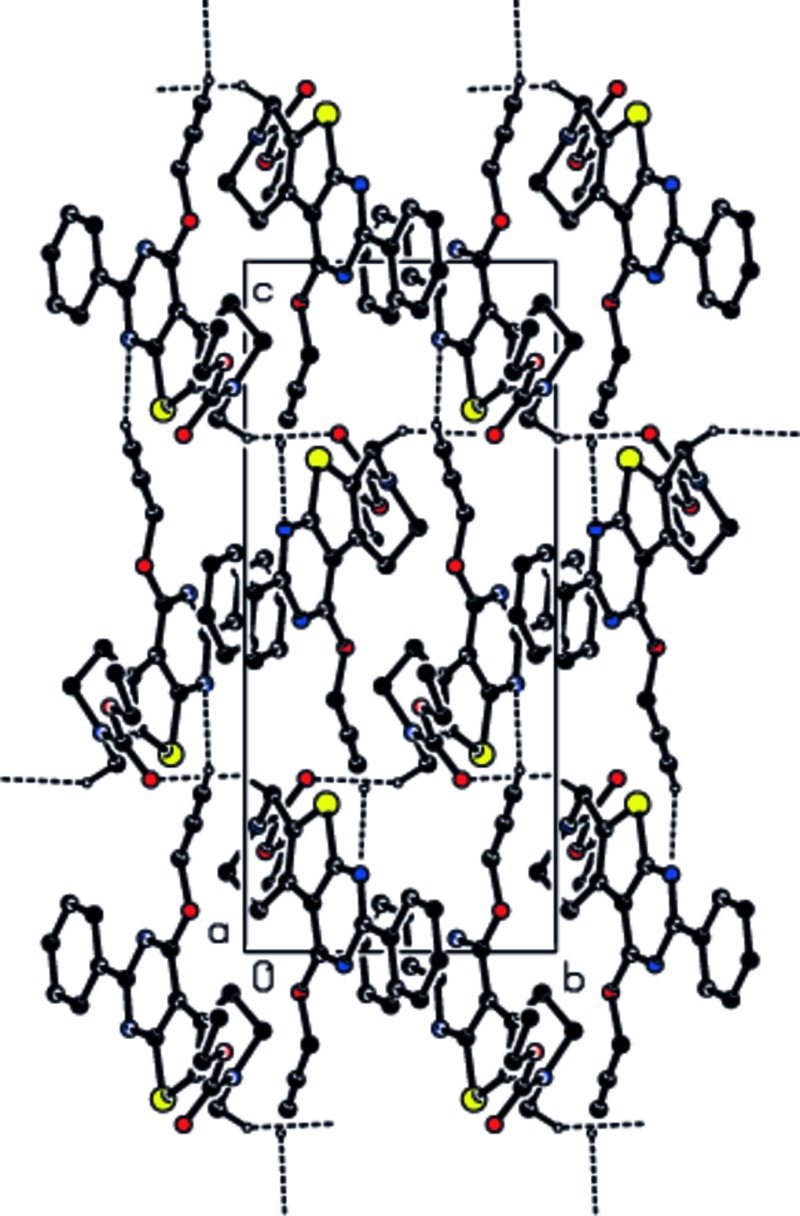
The molecular packing viewed down a axis. H atoms not involved in H bonding are omitted for clarity.

. DOI: 10.1107/S2056989015018447/tk5390fig1.tif
View of the title compound with the atom numbering scheme. Displacement ellipsoids for non-H atoms are drawn at the 50% probability level.

a . DOI: 10.1107/S2056989015018447/tk5390fig2.tif
The mol­ecular packing viewed down *a* axis. H atoms not involved in H bonding are omitted for clarity.

CCDC reference: 1429186

Additional supporting information:  crystallographic information; 3D view; checkCIF report

## Figures and Tables

**Table 1:** Hydrogen-bond geometry (, ) *Cg*1 and *Cg*4 are the centroids of the S1,C9C11/C15 and C1C6 rings, respectively.

*D*H*A*	*D*H	H*A*	*D* *A*	*D*H*A*
C14H14*A*O2^i^	0.97	2.44	3.294(2)	146
C21H21N2^ii^	0.93	2.55	3.418(2)	156
C12H12*B* *Cg*4^iii^	0.97	2.80	3.6643(17)	149
C19H19*A* *Cg*1^iv^	0.97	2.92	3.6736(18)	136

Symmetry codes: (i) 
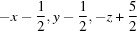
; (ii) 
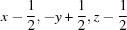
; (iii) 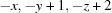; (iv) 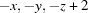.

## supplementary crystallographic information

### S1. Comment

Thieno[2,3-*d*]pyrimidines are a large group of heterocyclic compounds (Litvinov, 2004), and some of them showed antiviral (Nasr & Gineinah, 2002), antimicrobial (Bhuiyan *et al.*, 2005; Chambhare *et al.*, 2003), and antibacterial properties (Alagarsamy *et al.*, 2006). Fused tri- and tetracyclic thieno[2,3-*d*]pyrimidin-4-ones are synthesized by many methods and among them some compounds have fungicidal, antibacterial, and anti-inflammatory activities (Kapustina *et al.*, 1992). In this context, and following to our on-going study of bio-active molecules, we report here the synthesis and crystal structure of the title compound.

In the title compound (Fig. 1), the 5,6,7,8-tetrahydropyridine ring (N3/C11/-C15) adopts a half-chair conformation [the puckering parameters are Q_T_ = 0.4662 (14) Å, θ = 50.06 (17) ° and φ = 30.8 (2) °]. The fused-thieno[2,3-*d*]pyrimidine ring system (S1/N1/N2C7-C11/C15) is essentially planar (r.m.s. deviation = 0.001 Å) and forms a dihedral angle of 2.66 (6)° with the attached phenyl ring (C1–C6). The C8–O1–C19–C20, C13–N3–C16–O2 and N3–C16–O3–C17 torsion angles are −166.90 (12), −174.90 (13) and 179.78 (12)°, respectively. All bond lengths and angles in the title molecule are normal and comparable with those previously reported for related structures (Liu *et al.*, 2005; Ren *et al.*, 2006).

In the crystal, molecules are linked by C—H···O, C—H···N and C—H···π hydrogen bonds, forming a three dimensional network (Fig. 2 & Table 1).

### S2. Experimental

Propargyl bromide (1.1 g, 9 mmol) was added to a suspension of ethyl 4-hydroxy-2-phenyl-5,6-dihydropyrido[4',3':4,5]thieno[2,3-*d*]pyrimidine-7(8*H*)-carboxylate (1.07 g, 3 mmol) and K_2_CO_3_ (0.82 g, 6 mmol) in DMF (15 ml), and stirred at room temperature for 6 h. The excess solvent was evaporated to dryness *in vacuo*. The residue was diluted with water and then extracted with CH_2_Cl_2_ (3 *x* 30 ml). The combined organic extracts were dried over anhydrous Na_2_SO_4_, filtered and evaporated under reduced pressure to give colourless crystals in a sufficient quality for X-ray diffraction.

### S3. Refinement

All H atoms were positioned geometrically and constrained to ride on their parent atoms (C—H = 0.93–0.97 Å) with *U*_iso_(H) = 1.2 or 1.5 *U*_eq_(C). The (−2 3 10), (6 1 24), (5 11 4), (−3 1 17), (−14 8 5), (6 5 11), (11 3 8) and (−3 4 24) reflections were omitted owing to very bad agreement.

### Crystal data

**Table:**

C_21_H_19_N_3_O_3_S	*F*(000) = 824
*M**_r_* = 393.45	*D*_x_ = 1.396 Mg m^−^^3^
Monoclinic, *P*2_1_/*n*	Mo *K*α radiation, λ = 0.71073 Å
Hall symbol: -P 2yn	Cell parameters from 2782 reflections
*a* = 13.143 (2) Å	θ = 3.7–32.7°
*b* = 8.013 (2) Å	µ = 0.20 mm^−^^1^
*c* = 17.880 (2) Å	*T* = 296 K
β = 96.129 (14)°	Prism, colourless
*V* = 1872.3 (6) Å^3^	0.28 × 0.14 × 0.08 mm
*Z* = 4	

### Data collection

**Table:**

Agilent Xcalibur, Eos, Gemini diffractometer	6319 independent reflections
Radiation source: Enhance (Mo) X-ray Source	4721 reflections with *I* > 2σ(*I*)
Graphite monochromator	*R*_int_ = 0.032
Detector resolution: 16.0416 pixels mm^-1^	θ_max_ = 33.1°, θ_min_ = 3.1°
ω scans	*h* = −16→19
Absorption correction: multi-scan (*CrysAlis PRO*; Agilent, 2014)	*k* = −11→5
*T*_min_ = 0.834, *T*_max_ = 1.000	*l* = −25→26
14345 measured reflections	

### Refinement

**Table:**

Refinement on *F*^2^	0 restraints
Least-squares matrix: full	Hydrogen site location: inferred from neighbouring sites
*R*[*F*^2^ > 2σ(*F*^2^)] = 0.044	H-atom parameters constrained
*wR*(*F*^2^) = 0.117	*w* = 1/[σ^2^(*F*_o_^2^) + (0.0488*P*)^2^ + 0.4469*P*] where *P* = (*F*_o_^2^ + 2*F*_c_^2^)/3
*S* = 1.04	(Δ/σ)_max_ = 0.001
6319 reflections	Δρ_max_ = 0.35 e Å^−^^3^
254 parameters	Δρ_min_ = −0.31 e Å^−^^3^

### Special details

**Table:**

Geometry. Bond distances, angles *etc*. have been calculated using the rounded fractional coordinates. All su's are estimated from the variances of the (full) variance-covariance matrix. The cell e.s.d.'s are taken into account in the estimation of distances, angles and torsion angles
Refinement. Refinement on *F*^2^ for ALL reflections except those flagged by the user for potential systematic errors. Weighted *R*-factors *wR* and all goodnesses of fit *S* are based on *F*^2^, conventional *R*-factors *R* are based on *F*, with *F* set to zero for negative *F*^2^. The observed criterion of *F*^2^ > σ(*F*^2^) is used only for calculating -*R*-factor-obs *etc*. and is not relevant to the choice of reflections for refinement. *R*-factors based on *F*^2^ are statistically about twice as large as those based on *F*, and *R*-factors based on ALL data will be even larger.

### Fractional atomic coordinates and isotropic or equivalent isotropic displacement parameters (Å^2^)

**Table:**

	*x*	*y*	*z*	*U*_iso_*/*U*_eq_	
S1	−0.00878 (3)	0.26271 (5)	1.21398 (2)	0.0319 (1)	
O1	−0.08120 (7)	0.17676 (13)	0.94088 (4)	0.0274 (3)	
O2	−0.38389 (8)	0.19716 (14)	1.25060 (6)	0.0369 (3)	
O3	−0.45126 (7)	0.06635 (13)	1.14545 (5)	0.0325 (3)	
N1	0.06668 (8)	0.31874 (14)	0.98114 (5)	0.0249 (3)	
N2	0.11346 (8)	0.37095 (15)	1.11181 (6)	0.0276 (3)	
N3	−0.28451 (9)	0.03958 (16)	1.18227 (6)	0.0314 (3)	
C1	0.24352 (11)	0.48532 (17)	0.94671 (7)	0.0307 (4)	
C2	0.33262 (12)	0.5631 (2)	0.93051 (8)	0.0366 (4)	
C3	0.40123 (11)	0.6245 (2)	0.98760 (9)	0.0371 (4)	
C4	0.38043 (11)	0.6085 (2)	1.06113 (8)	0.0371 (4)	
C5	0.29176 (10)	0.5315 (2)	1.07789 (8)	0.0335 (4)	
C6	0.22226 (10)	0.46768 (16)	1.02092 (7)	0.0263 (3)	
C7	0.12892 (9)	0.38147 (16)	1.03956 (7)	0.0250 (3)	
C8	−0.01623 (9)	0.23971 (16)	0.99661 (6)	0.0235 (3)	
C9	−0.04163 (9)	0.21700 (16)	1.07030 (6)	0.0232 (3)	
C10	0.02839 (10)	0.28884 (17)	1.12482 (7)	0.0262 (3)	
C11	−0.12525 (9)	0.13753 (16)	1.10142 (6)	0.0242 (3)	
C12	−0.21248 (10)	0.04461 (17)	1.06005 (7)	0.0258 (3)	
C13	−0.26836 (11)	−0.05731 (18)	1.11502 (7)	0.0313 (4)	
C14	−0.19118 (10)	0.0880 (2)	1.22777 (7)	0.0316 (4)	
C15	−0.11712 (10)	0.15527 (17)	1.17729 (7)	0.0266 (3)	
C16	−0.37375 (10)	0.10798 (17)	1.19685 (7)	0.0283 (3)	
C17	−0.54903 (11)	0.1367 (2)	1.15901 (9)	0.0382 (4)	
C18	−0.62591 (13)	0.0838 (2)	1.09628 (10)	0.0491 (6)	
C19	−0.05720 (10)	0.20882 (18)	0.86513 (6)	0.0279 (3)	
C20	−0.14696 (11)	0.1659 (2)	0.81452 (7)	0.0336 (4)	
C21	−0.21842 (12)	0.1381 (3)	0.77141 (9)	0.0533 (6)	
H1	0.19760	0.44460	0.90780	0.0370*	
H2	0.34630	0.57400	0.88080	0.0440*	
H3	0.46110	0.67630	0.97650	0.0450*	
H4	0.42650	0.64990	1.09980	0.0440*	
H5	0.27830	0.52210	1.12770	0.0400*	
H12A	−0.18690	−0.02910	1.02330	0.0310*	
H12B	−0.25960	0.12300	1.03350	0.0310*	
H13A	−0.33400	−0.09310	1.09030	0.0380*	
H13B	−0.22870	−0.15630	1.12980	0.0380*	
H14A	−0.16170	−0.00790	1.25530	0.0380*	
H14B	−0.20610	0.17250	1.26390	0.0380*	
H17A	−0.54460	0.25750	1.16090	0.0460*	
H17B	−0.56870	0.09700	1.20660	0.0460*	
H18A	−0.69180	0.12800	1.10420	0.0740*	
H18B	−0.62930	−0.03590	1.09460	0.0740*	
H18C	−0.60620	0.12500	1.04950	0.0740*	
H19A	0.00070	0.14160	0.85410	0.0330*	
H19B	−0.04000	0.32550	0.85940	0.0330*	
H21	−0.27520	0.11590	0.73720	0.0640*	

### Atomic displacement parameters (Å^2^)

**Table:**

	*U*^11^	*U*^22^	*U*^33^	*U*^12^	*U*^13^	*U*^23^
S1	0.0328 (2)	0.0447 (2)	0.0173 (1)	−0.0006 (2)	−0.0014 (1)	−0.0032 (1)
O1	0.0298 (4)	0.0363 (5)	0.0155 (4)	−0.0005 (4)	−0.0002 (3)	−0.0024 (3)
O2	0.0381 (5)	0.0412 (6)	0.0326 (5)	−0.0020 (4)	0.0090 (4)	−0.0131 (4)
O3	0.0330 (5)	0.0359 (5)	0.0287 (4)	−0.0009 (4)	0.0033 (4)	−0.0053 (4)
N1	0.0258 (5)	0.0280 (5)	0.0201 (4)	0.0051 (4)	−0.0008 (4)	−0.0011 (4)
N2	0.0279 (5)	0.0330 (6)	0.0211 (5)	0.0039 (4)	−0.0013 (4)	−0.0016 (4)
N3	0.0328 (6)	0.0374 (6)	0.0243 (5)	0.0000 (5)	0.0051 (4)	−0.0073 (5)
C1	0.0387 (7)	0.0254 (6)	0.0279 (6)	0.0036 (5)	0.0028 (5)	−0.0028 (5)
C2	0.0459 (8)	0.0332 (7)	0.0326 (7)	0.0015 (6)	0.0125 (6)	−0.0016 (6)
C3	0.0333 (7)	0.0321 (7)	0.0469 (8)	0.0025 (6)	0.0090 (6)	0.0037 (6)
C4	0.0317 (7)	0.0403 (8)	0.0375 (7)	−0.0004 (6)	−0.0039 (6)	0.0015 (6)
C5	0.0310 (6)	0.0402 (8)	0.0283 (6)	0.0006 (6)	−0.0017 (5)	0.0014 (6)
C6	0.0280 (6)	0.0240 (6)	0.0264 (6)	0.0073 (5)	0.0002 (5)	−0.0005 (5)
C7	0.0260 (6)	0.0258 (6)	0.0225 (5)	0.0078 (5)	−0.0012 (4)	−0.0015 (4)
C8	0.0267 (6)	0.0248 (6)	0.0179 (5)	0.0075 (5)	−0.0023 (4)	−0.0019 (4)
C9	0.0251 (6)	0.0250 (6)	0.0186 (5)	0.0072 (4)	−0.0012 (4)	−0.0010 (4)
C10	0.0277 (6)	0.0317 (7)	0.0184 (5)	0.0063 (5)	−0.0018 (4)	−0.0008 (4)
C11	0.0272 (6)	0.0244 (6)	0.0203 (5)	0.0072 (5)	−0.0006 (4)	−0.0018 (4)
C12	0.0284 (6)	0.0275 (6)	0.0211 (5)	0.0052 (5)	0.0006 (4)	−0.0049 (4)
C13	0.0381 (7)	0.0292 (7)	0.0272 (6)	−0.0001 (6)	0.0064 (5)	−0.0071 (5)
C14	0.0351 (7)	0.0395 (8)	0.0204 (5)	0.0013 (6)	0.0035 (5)	−0.0010 (5)
C15	0.0287 (6)	0.0302 (6)	0.0200 (5)	0.0051 (5)	−0.0009 (4)	−0.0012 (5)
C16	0.0351 (7)	0.0268 (6)	0.0237 (5)	−0.0031 (5)	0.0066 (5)	−0.0002 (5)
C17	0.0355 (7)	0.0382 (8)	0.0408 (7)	0.0040 (6)	0.0034 (6)	−0.0029 (6)
C18	0.0413 (9)	0.0483 (10)	0.0556 (10)	−0.0036 (8)	−0.0050 (7)	−0.0020 (8)
C19	0.0304 (6)	0.0355 (7)	0.0173 (5)	0.0013 (5)	0.0006 (4)	−0.0012 (5)
C20	0.0339 (7)	0.0465 (8)	0.0203 (5)	0.0003 (6)	0.0028 (5)	−0.0047 (5)
C21	0.0351 (8)	0.0966 (16)	0.0278 (7)	−0.0078 (9)	0.0016 (6)	−0.0129 (8)

### Geometric parameters (Å, º)

**Table:**

S1—C10	1.7292 (14)	C11—C15	1.3568 (17)
S1—C15	1.7319 (14)	C12—C13	1.5253 (19)
O1—C8	1.3397 (15)	C14—C15	1.4968 (19)
O1—C19	1.4461 (14)	C17—C18	1.489 (2)
O2—C16	1.2163 (17)	C19—C20	1.4495 (19)
O3—C16	1.3393 (16)	C20—C21	1.171 (2)
O3—C17	1.4472 (18)	C1—H1	0.9300
N1—C7	1.3529 (16)	C2—H2	0.9300
N1—C8	1.3150 (16)	C3—H3	0.9300
N2—C7	1.3316 (17)	C4—H4	0.9300
N2—C10	1.3389 (18)	C5—H5	0.9300
N3—C13	1.4657 (18)	C12—H12A	0.9700
N3—C14	1.4505 (18)	C12—H12B	0.9700
N3—C16	1.3451 (18)	C13—H13A	0.9700
C1—C2	1.384 (2)	C13—H13B	0.9700
C1—C6	1.3921 (18)	C14—H14A	0.9700
C2—C3	1.379 (2)	C14—H14B	0.9700
C3—C4	1.377 (2)	C17—H17A	0.9700
C4—C5	1.380 (2)	C17—H17B	0.9700
C5—C6	1.3910 (19)	C18—H18A	0.9600
C6—C7	1.4767 (18)	C18—H18B	0.9600
C8—C9	1.4052 (16)	C18—H18C	0.9600
C9—C10	1.3918 (18)	C19—H19A	0.9700
C9—C11	1.4330 (17)	C19—H19B	0.9700
C11—C12	1.4949 (18)	C21—H21	0.9300
			
C10—S1—C15	90.73 (6)	C2—C1—H1	120.00
C8—O1—C19	116.38 (10)	C6—C1—H1	120.00
C16—O3—C17	114.33 (11)	C1—C2—H2	120.00
C7—N1—C8	117.57 (10)	C3—C2—H2	120.00
C7—N2—C10	114.61 (11)	C2—C3—H3	120.00
C13—N3—C14	114.45 (11)	C4—C3—H3	120.00
C13—N3—C16	125.49 (11)	C3—C4—H4	120.00
C14—N3—C16	119.01 (11)	C5—C4—H4	120.00
C2—C1—C6	120.37 (13)	C4—C5—H5	120.00
C1—C2—C3	120.45 (13)	C6—C5—H5	120.00
C2—C3—C4	119.53 (14)	C11—C12—H12A	110.00
C3—C4—C5	120.49 (14)	C11—C12—H12B	110.00
C4—C5—C6	120.62 (13)	C13—C12—H12A	110.00
C1—C6—C5	118.54 (12)	C13—C12—H12B	110.00
C1—C6—C7	121.25 (12)	H12A—C12—H12B	108.00
C5—C6—C7	120.21 (12)	N3—C13—H13A	109.00
N1—C7—N2	125.67 (11)	N3—C13—H13B	109.00
N1—C7—C6	116.61 (11)	C12—C13—H13A	109.00
N2—C7—C6	117.71 (11)	C12—C13—H13B	109.00
O1—C8—N1	120.10 (10)	H13A—C13—H13B	108.00
O1—C8—C9	116.89 (11)	N3—C14—H14A	110.00
N1—C8—C9	123.01 (11)	N3—C14—H14B	110.00
C8—C9—C10	113.43 (11)	C15—C14—H14A	110.00
C8—C9—C11	133.67 (11)	C15—C14—H14B	110.00
C10—C9—C11	112.89 (10)	H14A—C14—H14B	108.00
S1—C10—N2	122.89 (10)	O3—C17—H17A	110.00
S1—C10—C9	111.40 (10)	O3—C17—H17B	110.00
N2—C10—C9	125.70 (11)	C18—C17—H17A	110.00
C9—C11—C12	127.49 (10)	C18—C17—H17B	110.00
C9—C11—C15	111.09 (11)	H17A—C17—H17B	109.00
C12—C11—C15	121.42 (11)	C17—C18—H18A	110.00
C11—C12—C13	110.15 (10)	C17—C18—H18B	109.00
N3—C13—C12	111.52 (12)	C17—C18—H18C	110.00
N3—C14—C15	108.88 (10)	H18A—C18—H18B	109.00
S1—C15—C11	113.87 (10)	H18A—C18—H18C	109.00
S1—C15—C14	120.79 (9)	H18B—C18—H18C	109.00
C11—C15—C14	125.33 (12)	O1—C19—H19A	110.00
O2—C16—O3	123.30 (12)	O1—C19—H19B	110.00
O2—C16—N3	124.33 (12)	C20—C19—H19A	110.00
O3—C16—N3	112.37 (11)	C20—C19—H19B	110.00
O3—C17—C18	107.83 (13)	H19A—C19—H19B	108.00
O1—C19—C20	107.35 (11)	C20—C21—H21	180.00
C19—C20—C21	176.57 (18)		
			
C10—S1—C15—C11	0.63 (11)	C2—C3—C4—C5	0.1 (2)
C15—S1—C10—N2	179.24 (12)	C3—C4—C5—C6	0.5 (2)
C15—S1—C10—C9	0.26 (11)	C4—C5—C6—C7	178.35 (13)
C10—S1—C15—C14	179.45 (12)	C4—C5—C6—C1	−0.9 (2)
C19—O1—C8—N1	−3.14 (17)	C1—C6—C7—N2	−179.40 (12)
C8—O1—C19—C20	−166.90 (12)	C5—C6—C7—N1	−178.14 (13)
C19—O1—C8—C9	176.72 (11)	C1—C6—C7—N1	1.06 (18)
C17—O3—C16—N3	179.78 (12)	C5—C6—C7—N2	1.40 (19)
C16—O3—C17—C18	178.23 (12)	O1—C8—C9—C11	0.2 (2)
C17—O3—C16—O2	0.06 (19)	N1—C8—C9—C10	1.12 (19)
C8—N1—C7—C6	179.19 (11)	O1—C8—C9—C10	−178.73 (11)
C7—N1—C8—C9	−0.62 (19)	N1—C8—C9—C11	−179.97 (14)
C8—N1—C7—N2	−0.3 (2)	C8—C9—C10—N2	−0.8 (2)
C7—N1—C8—O1	179.22 (11)	C8—C9—C10—S1	178.11 (9)
C10—N2—C7—C6	−178.91 (12)	C8—C9—C11—C15	−177.42 (14)
C10—N2—C7—N1	0.58 (19)	C10—C9—C11—C12	−178.29 (13)
C7—N2—C10—S1	−178.78 (10)	C10—C9—C11—C15	1.50 (16)
C7—N2—C10—C9	0.1 (2)	C8—C9—C11—C12	2.8 (2)
C14—N3—C16—O2	−7.3 (2)	C11—C9—C10—N2	−180.00 (13)
C13—N3—C16—O2	−174.90 (13)	C11—C9—C10—S1	−1.04 (15)
C14—N3—C16—O3	172.98 (12)	C9—C11—C15—C14	179.93 (12)
C13—N3—C14—C15	46.00 (16)	C12—C11—C15—S1	178.50 (10)
C13—N3—C16—O3	5.37 (19)	C15—C11—C12—C13	−14.47 (17)
C14—N3—C13—C12	−64.08 (15)	C9—C11—C15—S1	−1.31 (15)
C16—N3—C13—C12	104.02 (15)	C9—C11—C12—C13	165.30 (13)
C16—N3—C14—C15	−122.93 (13)	C12—C11—C15—C14	−0.3 (2)
C6—C1—C2—C3	−0.2 (2)	C11—C12—C13—N3	44.25 (15)
C2—C1—C6—C7	−178.48 (13)	N3—C14—C15—S1	166.88 (10)
C2—C1—C6—C5	0.7 (2)	N3—C14—C15—C11	−14.4 (2)
C1—C2—C3—C4	−0.2 (2)		

### Hydrogen-bond geometry (Å, º)

Cg1 and Cg4 are the centroids of the S1,C9–C11/C15 and C1–C6 rings, respectively.

**Table:**

*D*—H···*A*	*D*—H	H···*A*	*D*···*A*	*D*—H···*A*
C1—H1···N1	0.93	2.49	2.8050 (19)	100
C5—H5···N2	0.93	2.47	2.7961 (19)	101
C13—H13*A*···O3	0.97	2.30	2.7085 (19)	104
C14—H14*A*···O2^i^	0.97	2.44	3.294 (2)	146
C14—H14*B*···O2	0.97	2.33	2.7509 (19)	105
C21—H21···N2^ii^	0.93	2.55	3.418 (2)	156
C12—H12*B*···*Cg*4^iii^	0.97	2.80	3.6643 (17)	149
C19—H19*A*···*Cg*1^iv^	0.97	2.92	3.6736 (18)	136

Symmetry codes: (i) −*x*−1/2, *y*−1/2, −*z*+5/2; (ii) *x*−1/2, −*y*+1/2, *z*−1/2; (iii) −*x*, −*y*+1, −*z*+2; (iv) −*x*, −*y*, −*z*+2.
