# Supplementary figures and images for: Crystal structure of ethyl 2-phenyl-4-(prop-2-yn-1-yl­oxy)-5,6,7,8-tetra­hydro­pyrido[4′,3′:4,5]thieno[2,3-d]pyrimidine-7-carboxyl­ate
Source: Acta Crystallogr E Crystallogr Commun. 2015 Oct 10;71(Pt 11):o836–7. doi: 10.1107/S2056989015018447 (PMC4645036; doi:10.1107/S2056989015018447)

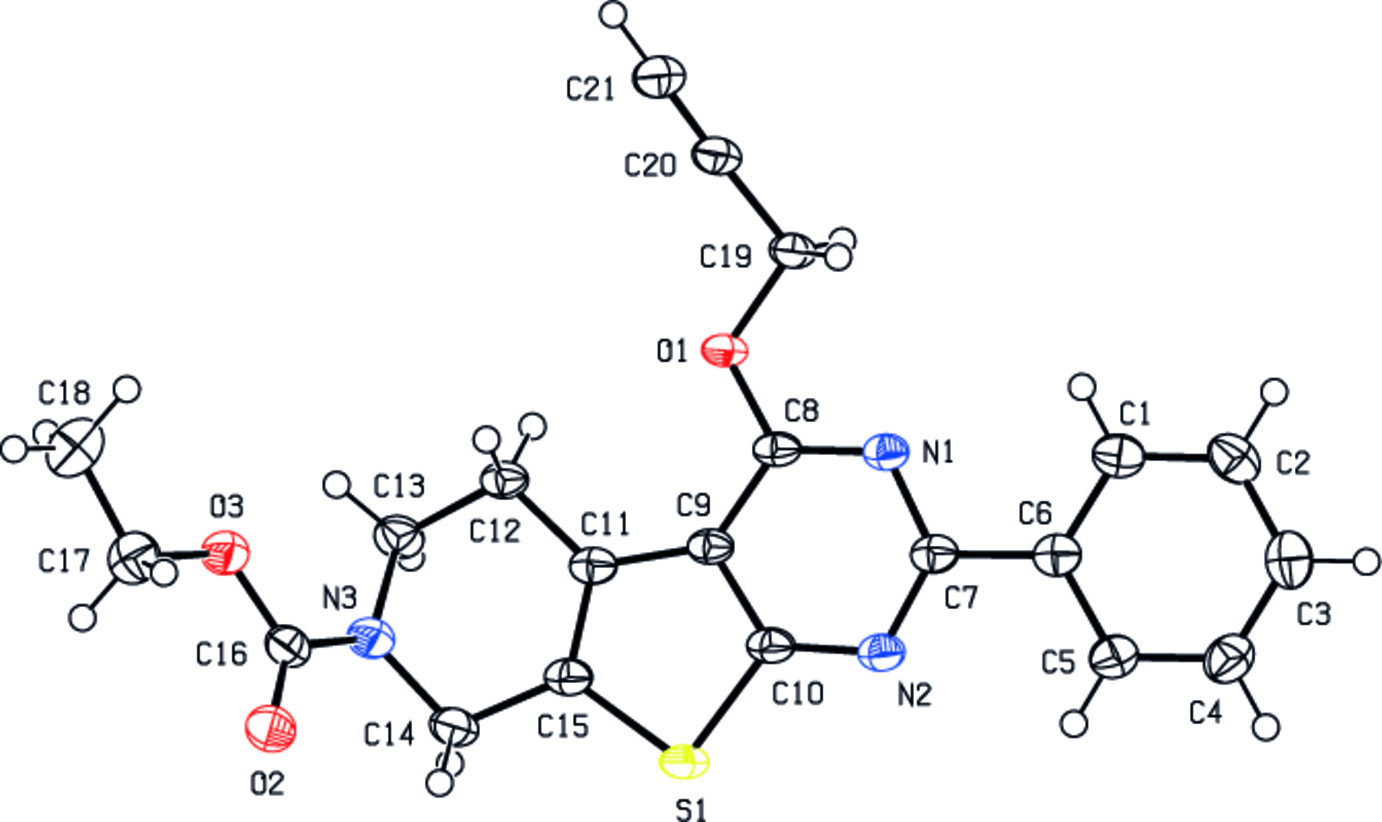

Supplement: Supplementary file 4 [file e-71-0o836-fig1.tif]

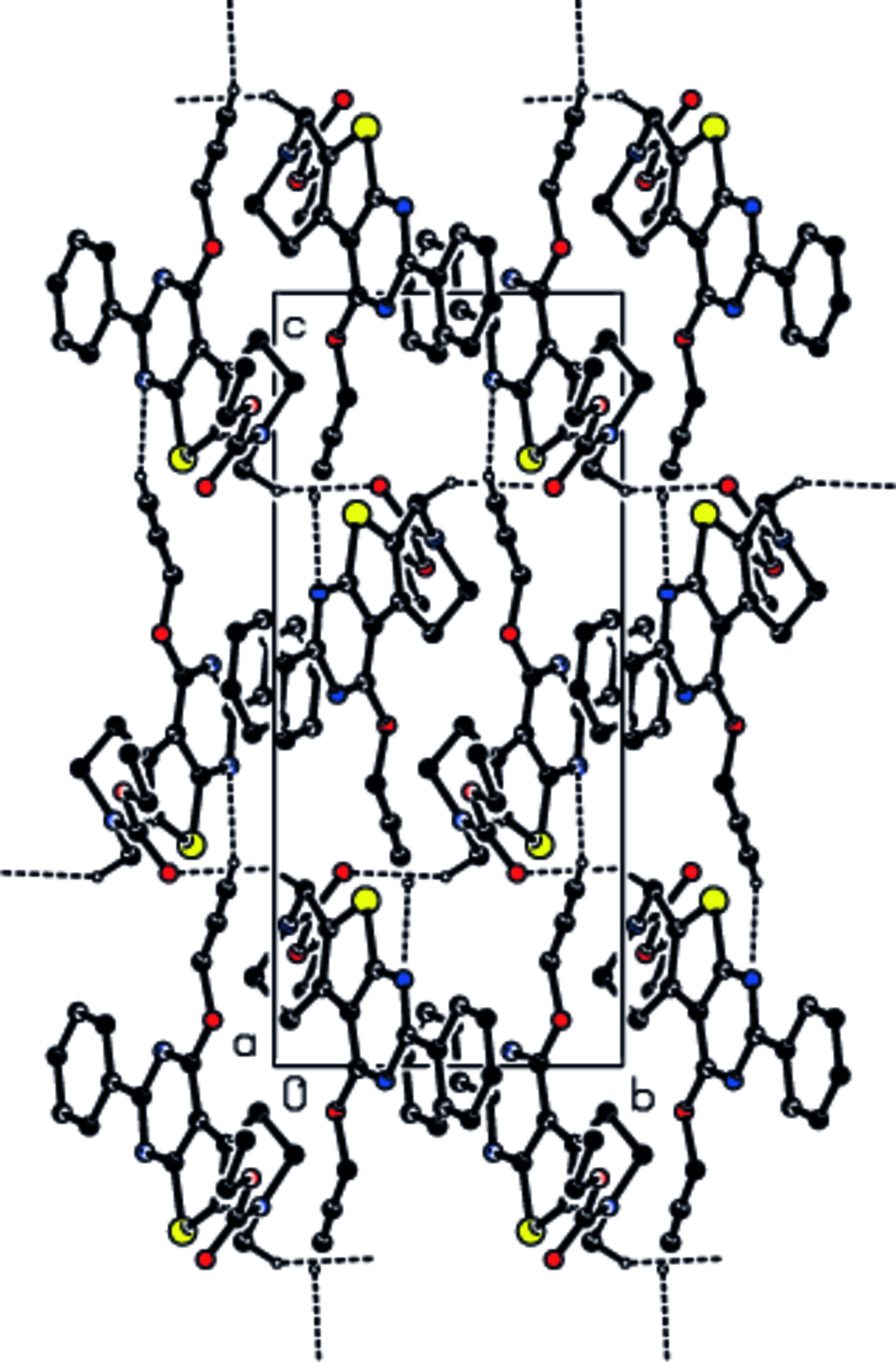

Supplement: Supplementary file 5 [file e-71-0o836-fig2.tif]
